# Anatomic grooved stem mitigates strain shielding compared to established total hip arthroplasty stem designs in finite-element models

**DOI:** 10.1038/s41598-018-36503-z

**Published:** 2019-01-24

**Authors:** Mark Heyland, Sara Checa, Daniel Kendoff, Georg N. Duda

**Affiliations:** 1 Julius Wolff Institute for Biomechanics and Musculoskeletal Regeneration, Charité — Universitätsmedizin Berlin, corporate member of Freie Universität Berlin, Humboldt – Universität zu Berlin, and Berlin Institute of Health, Berlin, Germany; 2 Berlin-Brandenburg Center for Regenerative Therapies, Charité — Universitätsmedizin Berlin, corporate member of Freie Universität Berlin, Humboldt – Universität zu Berlin, and Berlin Institute of Health, Berlin, Germany; 3 Berlin‐Brandenburg School for Regenerative Therapies, Charité — Universitätsmedizin Berlin, corporate member of Freie Universität Berlin, Humboldt – Universität zu Berlin, and Berlin Institute of Health, Berlin, Germany; 4 HELIOS Klinik Berlin-Buch, Berlin, Germany

## Abstract

Aseptic loosening remains a major problem for uncemented femoral components in primary total hip arthroplasty (THA). Ideally, bone adaptation after THA manifests minimally and local bone density reduction is widely avoided. Different design features may help to approximate initial, post-THA bone strain to levels pre-THA. Strain-shielding effects of different SP-CL stem design features are systematically analyzed and compared to CLS Spotorno and CORAIL using finite element models and physiological musculoskeletal loading conditions. All designs show substantial proximal strain-shielding: 50% reduced medial surface strain, 40–50% reduction at lateral surface, >120 µm/m root mean square error (RMSE) compared to intact bone in Gruen zone 1 and >60 µm/m RMSE in Gruen zones 2, 6, and 7. Geometrical changes (ribs, grooves, cross sections, stem length, anatomic curvature) have a considerable effect on strain-shielding; up to 20%. Combinations of reduced stem stiffness with larger proximal contact area (anatomically curved, grooves) lead to less strain-shielding compared to clinically established implant designs. We found that only the combination of a structurally flexible stem with anatomical curvature and grooves improves strain-shielding compared to other designs. The clinical implications *in vivo* of this initial strain-shielding difference are currently under evaluation in an ongoing clinical analysis.

## Introduction

Certain uncemented femoral stems (e.g. CLS Spotorno) in primary total hip arthroplasty (THA) show highly reliable outcomes with total implant survival around 94% and 86% after 10 and 22 years, respectively^1,2^. One of the last remaining issues of THA, with more than half of the late implant revisions, is aseptic loosening: the detachment of implant from bone in the absence of infection. Survivorship concerning femoral revision for aseptic loosening as the end point amounts to 93% at 22 years^2^, but there is an especially reduced total survival rate of some uncemented implants below 70% after 15 years in patients younger than 50 years^1^. A possible explanation for loosening might be the normal physiological bone remodeling process, and this is well founded because THA causes proximal bone unloading (stress- or strain-shielding, which represents the bypass transfer of load or deformation from bone to the implant) which often leads to a bone adaptation response, leading to reduced bone density in the proximal femur. This issue impacts especially young patients^3^.

Short stem or stemless implants have been proposed to reduce the stress-shielding^4^. Short stem implants show adequate survival rates at medium-term follow-up^5^. However, such short stem implants may be prone to subsidence, sometimes not even reducing bone resorption or leading to periprosthetic fractures^6–9^. Stemless implants with more reduced strain-shielding did not become widely accepted due to the increased probability of problematic implantation which is much more delicate without a guiding stem within the intramedullary canal^10^. Thus, geometrical features that enable physiological load transfer in standard implants remain the most promising approach to reduce strain-shielding.

However, it is unclear how different stem features in detail account for the unloading or if they would even allow regaining physiological bone strains. Numerous possibilities for a more physiological load transfer have been proposed such as anatomical stem curvature, reduction of material stiffness, and reduction of the stem cross-sectional area and length^11–16^. The present study concentrates on the distinct influence of stem curvature for improved fit and fill, the existence of grooves/ribs for increased contact surface and their inter-variable connection (i.e. changing two variables together) on bone strain compared to the intact bone within well-controlled physiological bone models. We hypothesize that a more anatomical stem curvature in combination with stem grooves would lead to a reduction of strain-shielding effects.

## Methods

**Figure 1:**
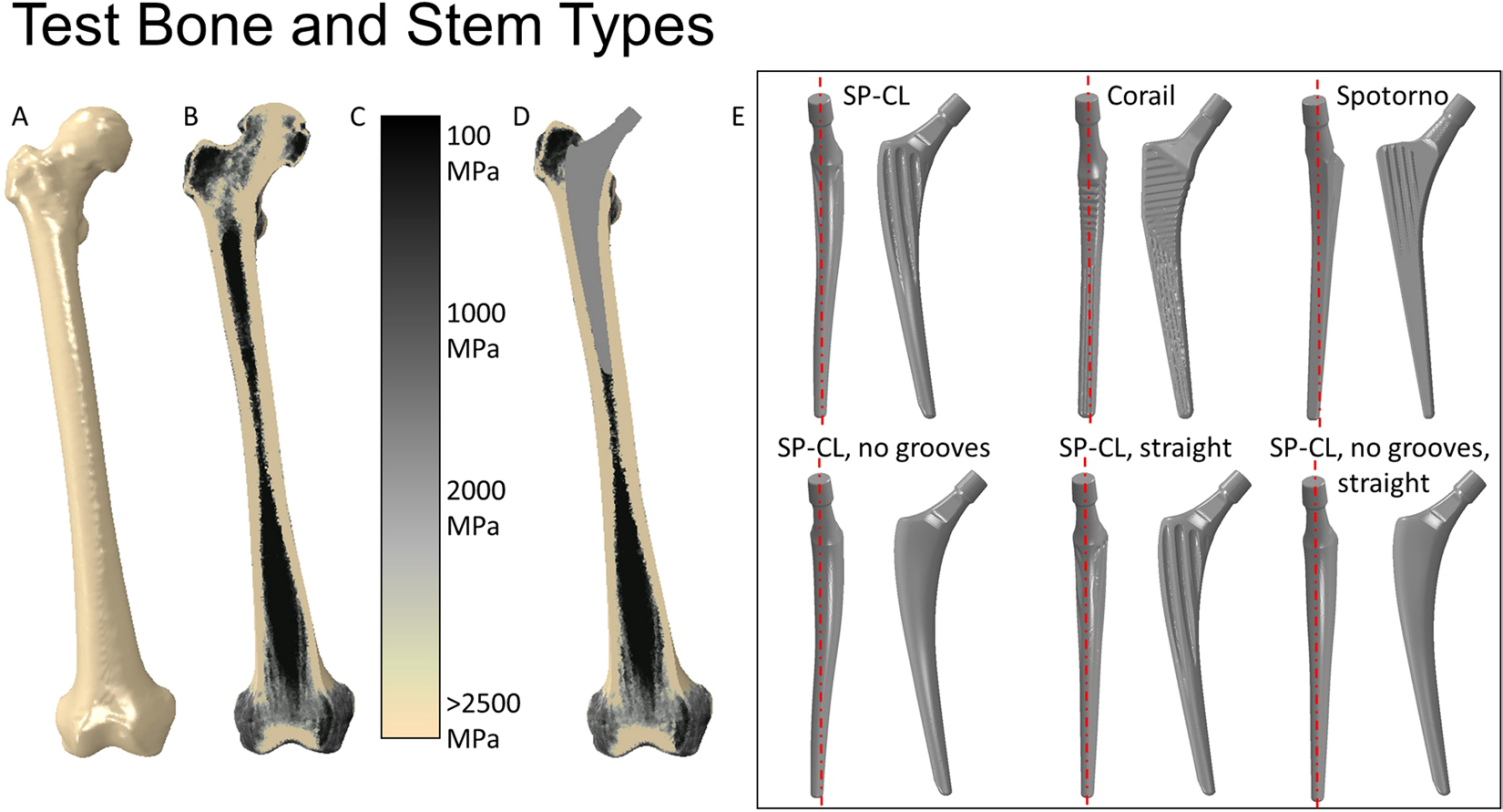
Overview of models: From left to right, (**A**) intact bone surface, (**B**) intact bone cut (coronal plane) with illustration of the material mapping approach, (**C**) legend showing Young’s modulus of the tissue, (**D**) illustration of THA in bone cut. On the right, (**E**) Tested implants seen from medial and from anterior (line in medial view to illustrate anatomically curved vs. straight stems).

Finite element (FE) models of an intact right femur of one representative female patient from a larger cohort who underwent THA in our clinic were created (Fig. 1A). No patients were directly involved in this study. Separate Institutional Review Board approval was not necessary as available, approved imaging data from our university hospital was used. A previous study collected this data, radiologic imaging was approved by the German Federal Office for Radiation Protection (Bundesamt für Strahlenschutz: Z 5 – 22462/2 – 2007-036). Patients signed that this data may be further processed for research, explicitly computer modelling. The bone geometry was extracted from quantitative computed tomography (qCT) images using image segmentation techniques (ZIBAmira 2013, Geomagic Studio 10). FE discretization was performed using meshes of more than 400,000 second-order ten-node tetrahedral elements per model, with a typical edge length of around 2 mm; which is known from previous studies to be sufficient for mesh convergence and fine material distribution that can differentiate cortical and cancellous bone areas^11,17,18^. This translates to element numbers of the stems ranging from 23,994 elements for a simple geometry to 142,192 for a complex surface geometry.

Material properties were assessed from qCT using the material mapping approach (Fig. 1B,C) according to previous studies^19,20^ as described before^21^. Pre-processing was performed using Abaqus CAE v6.12 to virtually remove the femoral head using a resection plane within the femoral neck (Fig. 1D) as applied hip joint load is validated for application at the tip of the neck of the femoral stem component. For the intact bone, load was applied at the corresponding position at the hip joint center. The size of the implants was chosen with the help of experienced surgeons. Implants were aligned according to standard surgical procedures, keeping the hip joint center (center of rotation) unchanged relative to the intact condition, which required slightly different implant lengths and CCD (Centrum-Collum-Diaphysis) angles.

The CORAIL and the CLS Spotorno implant stems represent two clinically well-tried examples of the most successful implants, which have proven excellent long-term results in cementless hip arthroplasty^22–25^. The CLS Spotorno stem allegedly manifests a more metaphyseal to diaphyseal load transfer while the CORAIL supposedly shows a more homogeneous load transfer to the bone. To analyze the effect of stem curvature and stem grooves, a new SP-CL design is systematically analyzed and compared to the CLS Spotorno and CORAIL designs. The investigated implants (Fig. 1E) are:

- CORAIL Collarless (DePuy Synthes, Johnson & Johnson Medical Devices, New Brunswick, USA), size 13, 135° CCD, L = 155 mm (straight, numerous small grooves)
- CLS Spotorno (Zimmer Biomet Holdings, Inc., Warsaw, USA) size 9, 135° CCD, L = 150 mm (straight, large ribs)
- SP-CL (Waldemar Link, Hamburg, Germany) size 10, 126° CCD, L = 160 mm (anatomically curved, mild grooves)

Variations of the SP-CL that are not manufactured by the company and only exist for the purposes of this study:

- SP-CL without grooves (smooth surface)
- SP-CL straight (with mild grooves)
- SP-CL straight without grooves.

The SP-CL feature variations enable direct comparison of the effects of stem curvature (indirectly gap fit and fill), grooves (larger surface, undercuts), and the interplay of different features. The relative cross-sectional areas of the implants are compared in Table 1.

**Table 1:** Relative cross-sectional areas of the different placed implants from proximally to distally with corresponding positions.

*Length [%]*	*Relative cross-sectional area [in % of CORAIL at 20% length]*					
	CORAIL	CLS Spotorno	SP-CL	SP-CL without grooves	SP-CL straight	SP-CL straight without grooves
*10*	**35**	39	41	41	41	41
*15*	92	**49**	112	111	113	113
*20*	**100**	113	103	119	103	119
*25*	92	94	**76**	103	**76**	103
*30*	78	78	**65**	91	**65**	91
*40*	57	**51**	54	69	54	69
*50*	42	**35**	42	49	42	49
*60*	36	**28**	34	35	34	34
*70*	31	**22**	27	27	27	27
*80*	26	**17**	21	21	21	21
*90*	22	**12**	16	16	16	16

Smallest cross-sections are marked in bold. SP-CL shows larger cross-sections than the other implants more proximally, but exhibits much less area from about 20–40% of the length in cranial-caudal direction.

**Figure 2:**
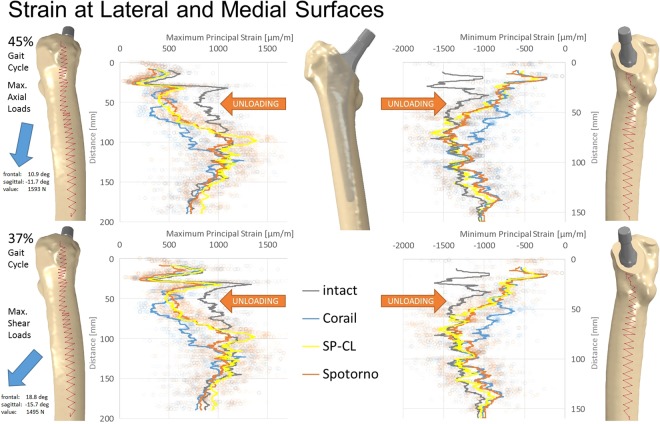
Maximum/minimum principal strain along path at the lateral (left) and medial (right) femoral surface at 45% (top) and 37% (bottom) of the gait cycle for different stem designs (Corail, SP-CL, Spotorno) and the intact bone. Please observe the dominant hip joint load on the left and their orientation pointing laterally, posteriorly, and caudally. Cranial-caudal path summarized with moving average, single measurement points shown as transparent circles demonstrating a large variance with position.

Material modulus of elasticity for metal titanium components was set at 110 GPa, Poisson ratio 0.3. The models were loaded with concentrated forces derived from a validated musculoskeletal model^26^; similar loads were used in finite element models before and are detailed there^21^. The main loading vector value and orientation at the femoral/implant head are shown in Fig. 2. The internal muscle and joint loads correspond to 37% and 45% of the gait cycle of the specifically chosen patient, using input data as measured in the gait lab with this specific patient. These time-points in the gait cycle are characteristic for the maximum loads in the horizontal plane (anterior-posterior, medial-lateral during one-legged stance, i.e. shear) and the maximum longitudinal loads (caudal-cranial, maximum ground reaction force and maximum hip force) respectively (Fig. 2, left).

Empirically realistic musculoskeletal boundary conditions were implemented^27^ that constrain a node at the knee centre in three translational degrees of freedom (DOFs). Another node, where the hip contact force was applied, was constrained in two DOFs such that this node could only deflect along an axis towards the knee centre. The sixth DOF was constrained at a node on top of the distal lateral epicondyle. The subject-specific loading conditions and physiological boundary constraints are essential because they lead to inter-patient variations in bone adaptation patterns^28^. At the bone-implant interface, initial tangential sliding and friction with a coefficient of 0.5 was considered until contact occurred. Then a normal, uniform penalty contact was modeled without separation (bony on-growth) where contact to bone was expected (i.e. where compact bone neighbors the implant or up to the end of grooves). While the contact interaction properties were the same for all implants as we expected bony on-growth, the contact surface varied based on the extent of grooves or coated areas. With the given model geometries, the stem tips simply float in the canal, so that at the distal implant tip contact was neglected.

After quasi-static analyses, maximum principal strains at the lateral surface and minimum principal strains at the medial surface and along internal paths were evaluated using Abaqus CAE v6.12 for post-processing. The strain values are sensitive to the exact measurement position (Fig. 2) and were therefore averaged (moving average) along the cranial-caudal direction over a small area (anterior-posterior extent shown with paths in Fig. 2). The internal paths were created through medio-lateral adjustment of the surface paths of 5 mm proximally and 2 mm distally towards the bone center.

The strain deviation to intact as root mean square error of maximum absolute principal strain per Gruen zone was computed using ZIBAmira 2013 through resampling and interpolation. To illustrate the load transfer at the bone-implant interface, contact pressure at the implant surface was evaluated for the different stem designs.

Model validation was performed by: (1) comparison of predicted strains (Fig. 2) to *in vivo* strain gauge measurements at the proximal lateral intact femur^29^ and (2) femoral head deflection and deflection at the mid-shaft as measured *in vivo* in a radiological study directly from 2D-X-rays during one legged stance^30^.

## Results

Predicted strains in the proximal lateral region of the intact femur were similar to those measured experimentally (model: 1057–1098 µm/m, experimental: 1013–1454 µm/m)^29^. Deflection of the femoral head was 0.93–1.09 mm and 1.72–1.91 mm at the femoral shaft for the intact femur, which is consistent with published results^30^.

**Figure 3:**
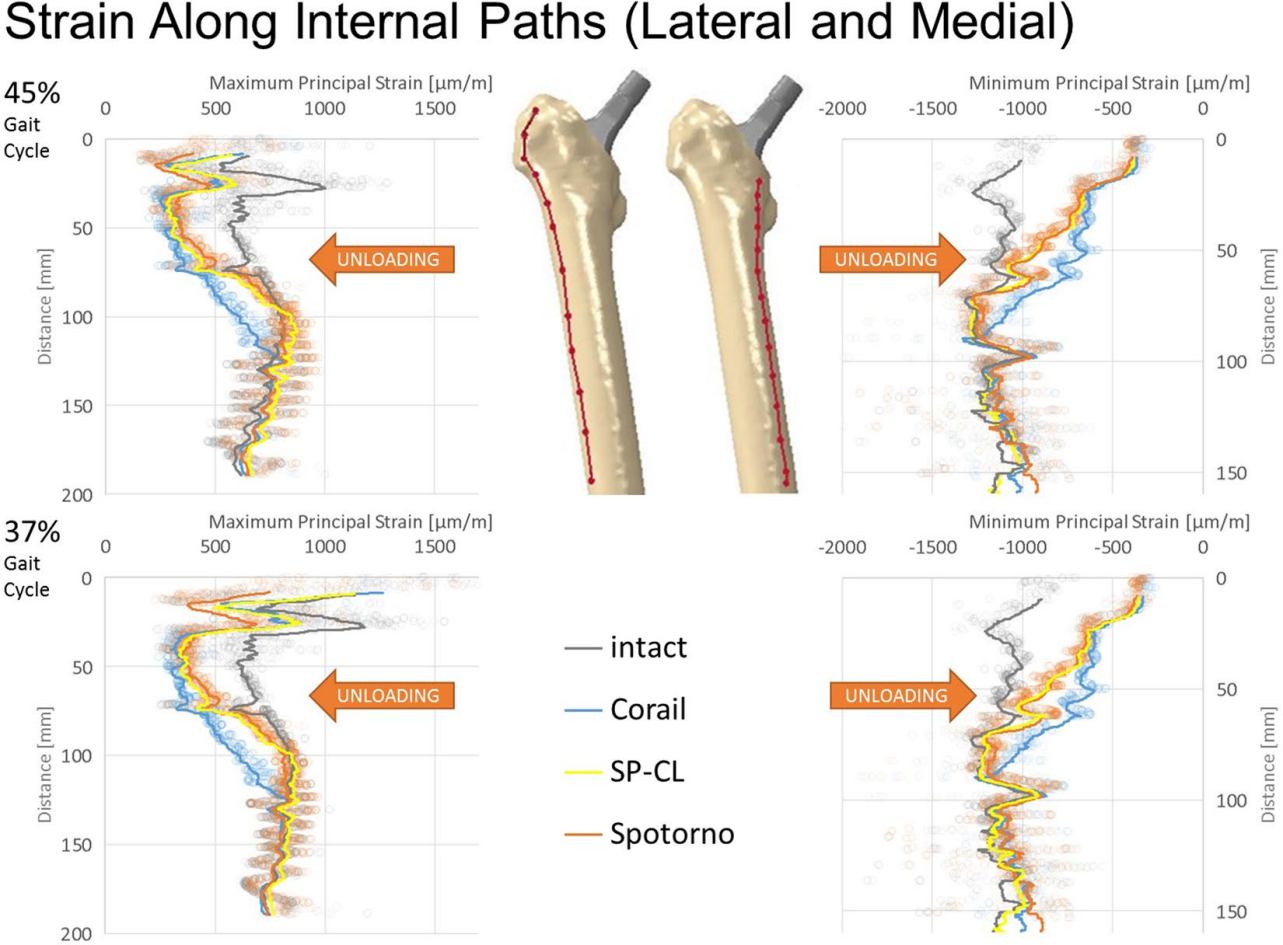
Maximum/minimum principal strain along internal path (AP-view showing depth) of the lateral (left) and medial (right) femoral bone at 45% (top) and 37% (bottom) of the gait cycle for different stem designs and the intact bone. Cranial-caudal path summarized with moving average, single measurement points shown as transparent circles demonstrating variance with position.

**Figure 4:**
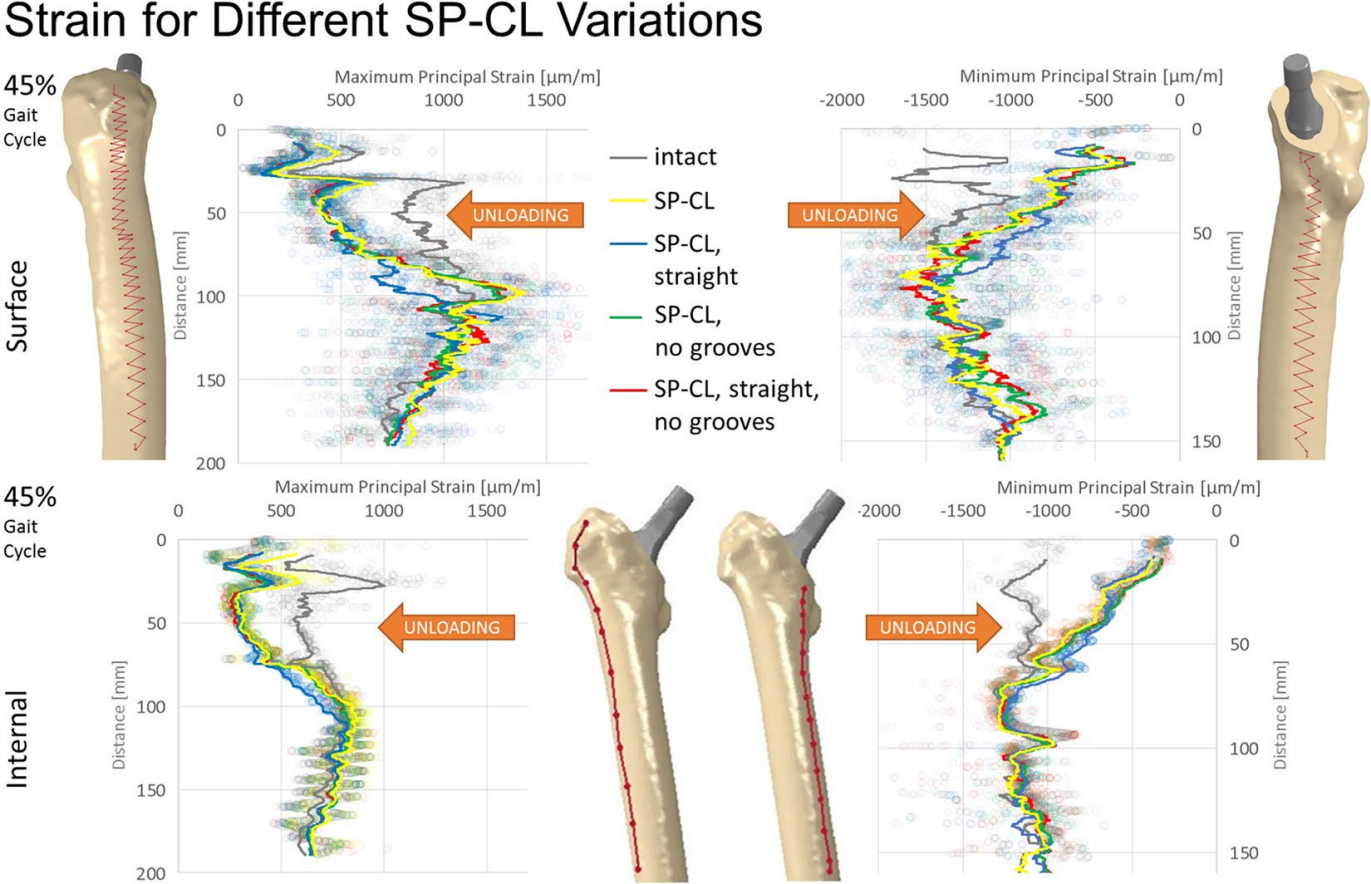
Maximum/minimum principal strain along surface path (top) and internal path (bottom) of the lateral (left) and medial (right) femoral bone at 45% of the gait cycle for different SP-CL design features and intact bone. Cranial-caudal path summarized with moving average, single measurement points shown as transparent circles demonstrating large variance with position.

**Figure 5:**
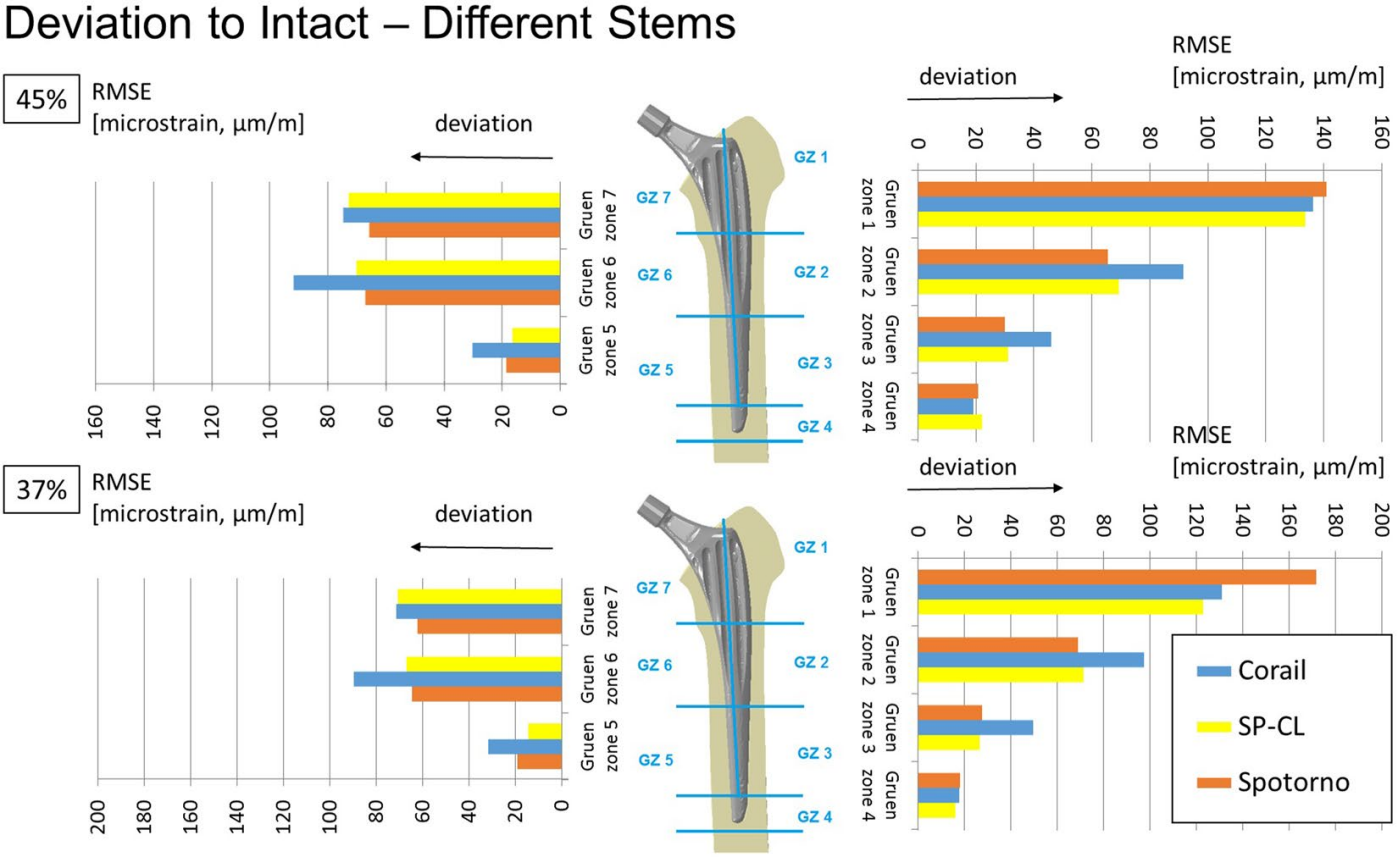
Deviation to intact (RMSE = root mean square error) per Gruen zone (GZ) at 45% (top) and 37% (bottom) of the gait cycle for different stem designs.

**Figure 6:**
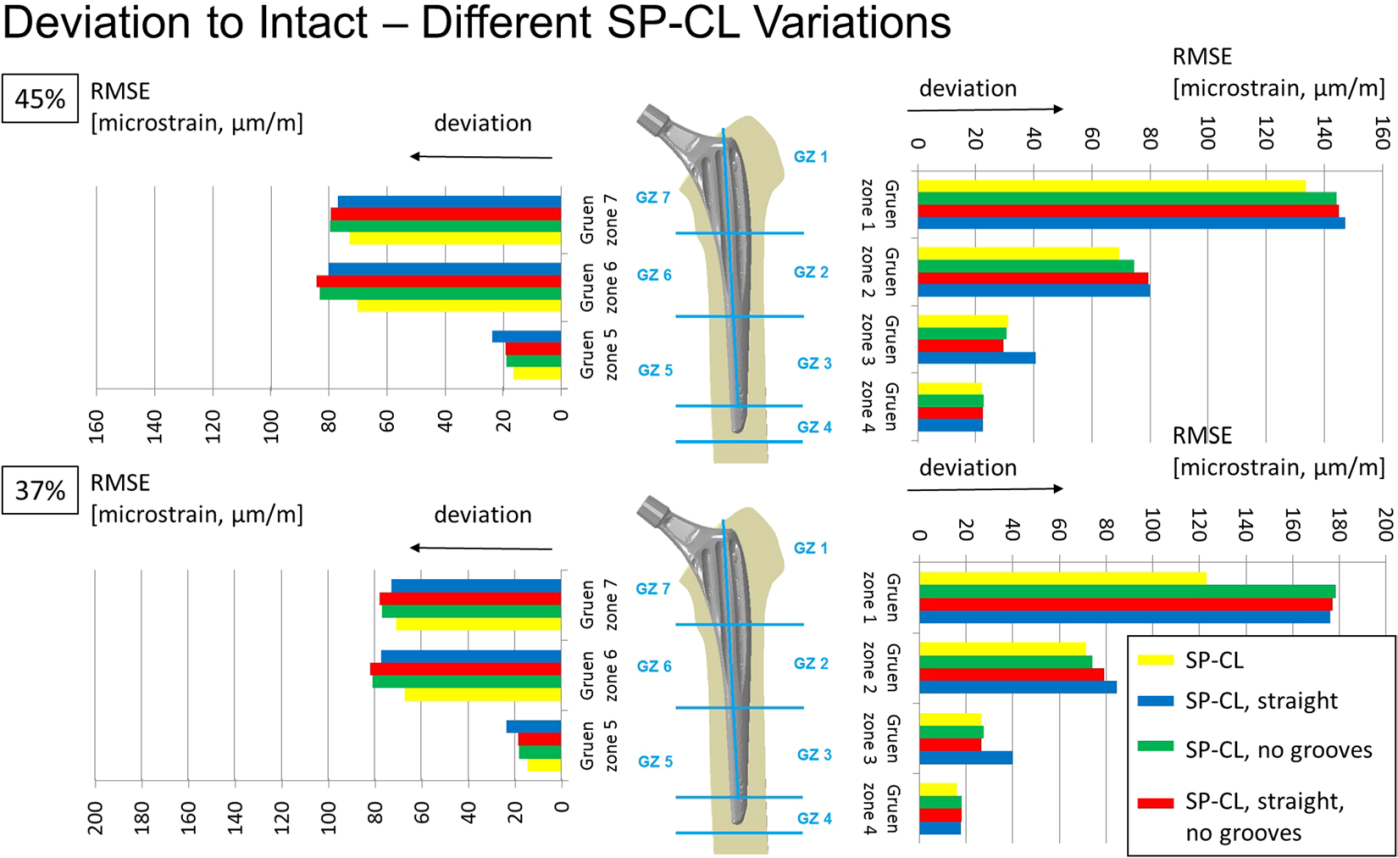
Deviation to intact (RMSE = root mean square error) per Gruen zone (GZ) at 45% (top) and 37% (bottom) of the gait cycle for different SP-CL design features.

All stem designs generally showed similar qualitative proximal strain-shielding at the bone surface (reduced strain compared to intact), between 40–50% laterally and in parts higher than 50% medially. More distally, strain-shielding is minimal and even an overstraining can be observed at the distal third of the implants (Fig. 2). The differences of strain-shielding for different stem designs are mostly moderate compared to the deviation to intact (Figs 3–6). The highest deviations to intact (Figs 5 and 6) are observed in Gruen zone 1 with over 120 microstrain root mean square error (RMSE) for all stem designs and over 60 microstrain RMSE in Gruen zones 2, 6, and 7. Gruen zones 4 and 5 show small deviations to intact of about 20 microstrain RMSE. Strain-shielding also reaches the volume inside the bone (Fig. 3). Strain values in intact bone versus bone with implant seem to converge distally (Fig. 3). Within the proximal bone tissue, there are still consistently high strains in intact bone and the strain-shielding caused by the implants remains similar to that at the bone surface.

**Figure 7:**
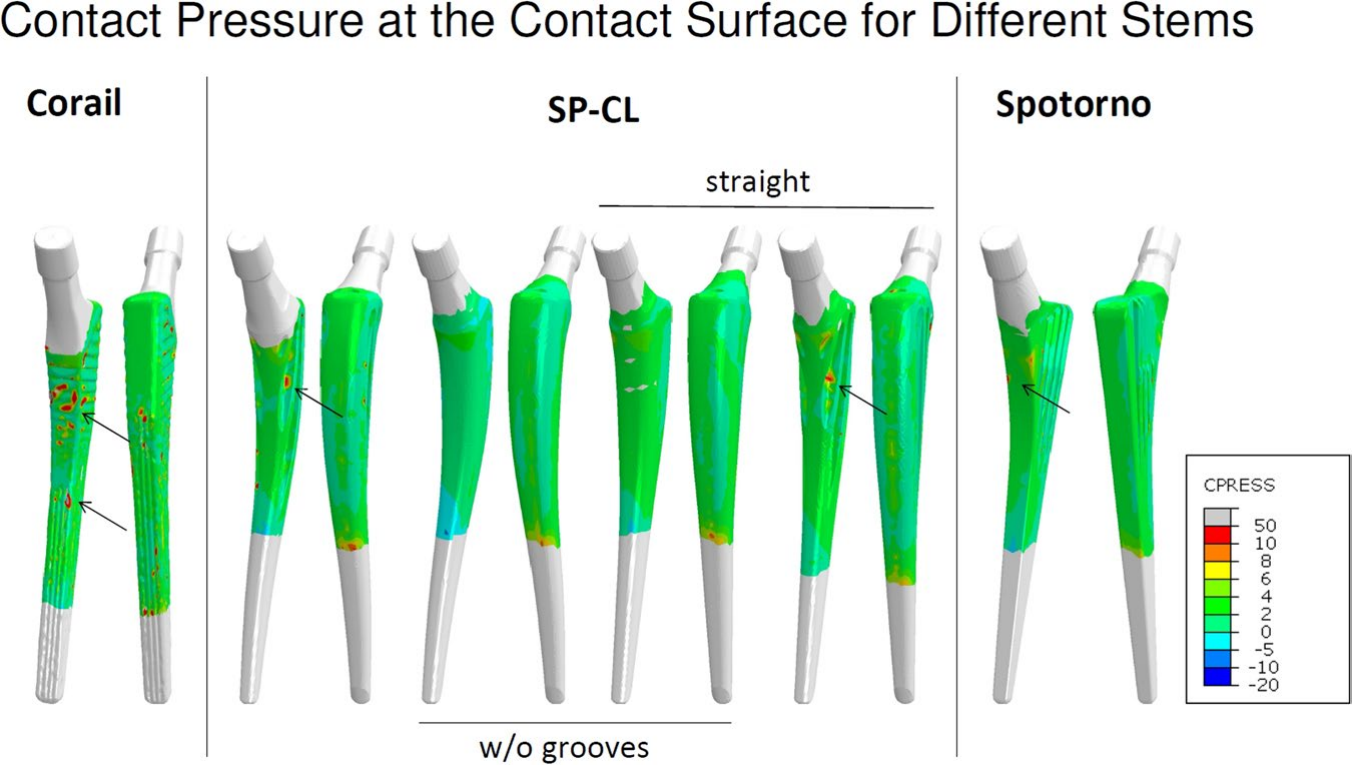
Load transfer at the bone-implant interface shown as contact pressure (MPa) at the implant surface at 45% of the gait cycle for different stem designs.

**Figure 8:**
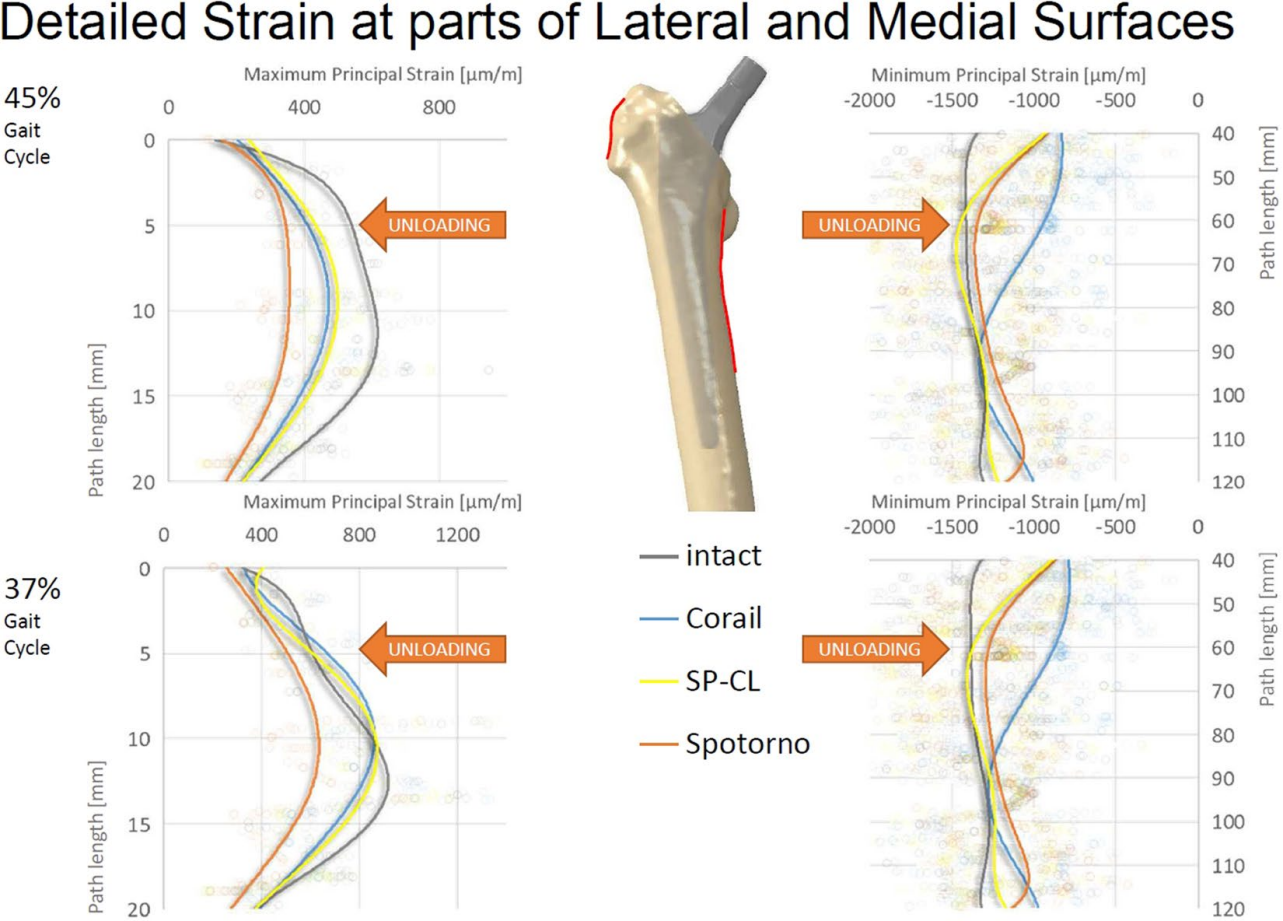
Detail of Fig. 2 with maximum/minimum principal strain along path at lateral (left) and medial (right) femoral surface at 45% (top) and 37% (bottom) of the gait cycle for different stem designs (Corail, SP-CL, Spotorno) and intact bone. Cranial-caudal path summarized with 6th degree polynomic functions, single measurement points shown as transparent circles demonstrating large variance with position.

The load transfer from the stem to the bone is not homogeneous (Figs 7 and 8). Especially the straight stems with grooves or ribs show marked (arrows in Fig. 7) stress concentrations. The CLS Spotorno stem manifests a relatively focused proximal diaphyseal load transfer compared to the other tested implants (Figs 7 and 8) with its reduced cross-sectional area below 40% of the length from proximally to distally and thus metaphyseal bracing (Table 1). In contrast, the CORAIL shows a more widely distributed, but not homogeneous load transfer to the bone, with a concentration even more distally than in the CLS Spotorno (Fig. 7). The SP-CL shows more even load transfer with less focus of stress. In the detailed surface strain (Fig. 8), SP-CL and CORAIL are close to intact strain very proximally lateral especially for high shear loads (37% of the gait cycle), while more distally (medial) at the diaphysis, SP-CL and CLS Spotorno are closer to intact strain. An appreciable strain-shielding of the CLS Spotorno in Gruen zone 1 (Fig. 5 at 37% of the gait cycle, Fig. 8 left) was observed with about 40% reduced surface strain versus intact (>350 microstrain at the proximal surface for any load, >170 microstrain RMSE in Gruen zone 1 at 37% of the gait cycle). In contrast, the SP-CL and CORAIL showed on average 20% to 25% reduced strain versus intact in the proximal lateral surface, respectively (Fig. 8 left: ca. 120–150 microstrain for 45% of the gait cycle, <100 microstrain for 37% of the gait cycle, Fig. 5: 120–130 microstrain RMSE in Gruen zone 1). CORAIL leads to a reduction of 20% medial surface strain versus intact in Gruen zones 2 and 6 (Figs 5 and 8 right), while CLS Spotorno leads to only about 11% reduction and SP-CL exhibits on average medially 4% reduction of strain with even some small zones of overloading. Overall, the strain-shielding differences among different stem designs are maximal in the proximal part, but generally remain below 20%.

Using the SP-CL implant as a template, the effects of individual changes to stem design are shown in Fig. 6. Interestingly, while singular changes resulted in small strain changes (up to about 10 microstrain RMSE), multiple marginal changes to stem design led to explicit changes (>40 microstrain) in strain-shielding. This can be observed as an improvement towards the intact case for SP-CL (curved anatomically and with grooves, Figs 6 and 8) and away from intact strain for Spotorno (straight, bulky top with ribs) for the load at 37% of the gait cycle (high rotational loads) in Gruen zone 1 (Figs 5 and 8).

## Discussion

This study uses a novel THA stem (SP-CL) as an example to illustrate the effect of various design features (anatomical shape and ribs/grooves) on the amount of strain-shielding in the femur. The analyses set out to investigate the distinct influence of the stem design features and their inter-variable connection on bone strain compared to the intact bone within a physiological model. Our results show that a novel implant incorporating anatomical shape and grooves (SP-CL) leads to less strain-shielding than well-established total hip replacements (CORAIL and CLS Spotorno).

Inaba, *et al*.^31^ reported peri-prosthetic bone loss 6 months postoperatively in the proximal and middle zones (Gruen zones 1, 2, 6, and 7) for different femoral stem designs (VerSys Fiber Metal MidCoa, SL-Plus, Accolade TMZF). Bone strain reductions with a CLS Spotorno stem have been found in experimental studies in the proximal femur with a mean difference on the medial side at the calcar of −65%, laterally −72%, and more distally up to −24% when compared to the intact femur, while the very distal strain showed only minor changes^32^. Such experimental *in vitro* studies, where a 50–64% drop in proximal surface strain, and slight distal changes of 4–14% of strain increase were recorded^33^, agree qualitatively with clinically observed volumetric bone density changes after THA. Szwedowski, *et al*.^28^ report for 12 months post-THA clinically measured BMD changes of −9.2 to −17.2% in Gruen zone 1, −15.9 to −33.6% in Gruen zone 7, and −12.9 to +4.9% in the more distal Gruen zones 3–5 for 3 patients with an uncemented Zimmer Alloclassic stem. Those values of BMD changes generally agree with our results, but the exact values for experimentally measured strain-shielding vary in different studies due to inconsistent loading and measurement sensor positioning^34^. With our approach, a consistent measurement under controlled physiologic-like boundary conditions is possible at exactly corresponding positions (Fig. 2).

Our study, coherently with the literature, finds unloading at a relevant level for remodeling at the proximal femur for all tested implant variations. Strain-shielding higher than 100 microstrain has been reported to induce periprosthetic bone loss, which can be lowered to about 50 microstrain through estrogen influence^35^. For our models (Figs 5–7), this suggests serious initial remodeling activity in Gruen zone 1 and moderate activity in Gruen zones 2, 6 and 7 for all stem designs. There is a wide distribution of strain and single elements show deviations well above 100 microstrain even if RMSE stays below this threshold. Our results confirm that even the successful implants CORAIL and CLS Spotorno show unloading of 50% at the proximal femoral surface (Fig. 2). The reported strain-shielding values from the literature agree well with the high proximal medial strain-shielding at the surface that we have found^32^. In addition, our results highlight that the local variation of strain-shielding remains high, even in separate Gruen zones, as we have seen the highest strain-shielding RMSE in Gruen zone 1, and not 7 (Fig. 5). Our computational results indicate that internally, within the bone, the absolute strain differences to intact bone (unloading) tend to get slightly smaller and more uniform, i.e. less variance of strain distribution (Fig. 3). However, the level of strain reduction over 60 microstrain RMSE (with many singular element deviations over 100 microstrain) remains overall still relevant for remodeling in Gruen zones 1, 2, 6, and 7 (Fig. 5).

The CLS Spotorno showed proximally (Figs 5 and 8) about 15–20% higher strain-shielding versus CORAIL and SP-CL respectively. However, more distally, the CORAIL implant (Figs 5 and 8) showed about 10–15% more strain-shielding than CLS Spotorno and SP-CL, respectively. These initial deviations in strain-shielding seem to play an important role for postoperative BMD loss of the proximal femur as Inaba, *et al*.^31^ found clear differences in proximal bone remodeling among 3 commonly used uncemented stems different from those in our study. The SP-CL stem seems to adopt the strengths of CLS Spotorno and CORAIL and, although it does not stand out in a certain area compared to the successful stem designs, the SP-CL stem ultimately leads to least overall deviation of strains compared to the intact femur of the tested stems in this study. Thus, we would assume a clinical relevance comparable to the differences of outcome seen with different implants such as CORAIL and CLS Spotorno in respect to aseptic loosening. Those differences in strain-shielding might be one of possible explanations (together with wear-induced inflammation and altered bone remodeling) to the up to 10% of additional implant survival after 2 decades, with 86% survival for CLS Spotorno after 22 years^2^ versus 96% for CORAIL after 23 years^24^.

Surprisingly, individual features such as straight against curved design, ribs, and small and larger grooves only showed a mild influence on the strain deviation to intact (Figs 4–6) and are rather comparable for a similar position of the implant and comparable load. The general pattern of unloading does not vary with the investigated loads (as can be compared for the maximum longitudinal loads at 45% of the gait cycle and the maximum shear loads at 37% of the gait cycle tested here), but only in their distinct amount of deviation to intact. This suggests that unloading of bone and resulting bone adaptation represents a general effect or problem that can only mildly be altered and reduced with adapted loading. Based on our observations in varying shear and axial loading in this study, we extract that the general pattern of unloading of the bone remains rather similar even for a broader range of activities (and is not activity-specific) and thus we would not expect a drastic difference in other activities such as sports or rehabilitation activities. Adapted patient activities and certain activities such as sports or rehabilitation are not likely to solve the problem of detrimental bone adaptation. However, in our investigation, we have found a susceptibility of the CLS Spotorno towards higher strain-shielding during torsional loading around the femoral/stem shaft compared to CORAIL or SP-CL, possibly due to the square cross-section of the CLS Spotorno. Thus, lifestyle or activity profile of patients may play a small, but contributing role for aseptic loosening of certain implants.

Decking, *et al*.^36^ report that a rectangular straight stem led to a reduction of strains below the calcar −73%, and below the greater trochanter −61% while a (mostly smooth) curved stem led to a reduction of major principal strains −43% below the calcar and −69% below the greater trochanter. In our models, the combination of anatomically curvature and grooves (larger surface area) showed an equivalent strain-shielding or even slight improvement of load transfer proximally lateral compared to the already successful straight stem designs (Figs 2, 5 and 6) with an even stress transfer laterally, but not medially (Fig. 7). Aamodt, *et al*.^37^ compared the strain-shielding for an anatomical stem (without grooves) and a gap-filling stem based on cross-sectional CT scans. They found that principal compressive strain at the calcar was reduced by 90% for an anatomical stem and 67% for a gap-filling stem, while medially, at the level of the lesser trochanter, the corresponding figures were 59% and 21%. This underlines the importance of geometric match or sufficient contact area between femoral canal and stem even when anatomic stems are used. The exact long-term consequences of this mildly, but distinctly enhanced stimulation with a stem that fits and features large-area-contact to the bone (i.e. anatomically curved and ribs or grooves) cannot be precisely assessed with the current methods. A remodeling algorithm may extrapolate the initial strain differences to future density changes. However, differences of BMD changes of different stems suggest that a magnitude of about 10% BMD change after 3 years could be realistic^31^. The local differences in strain-shielding between well-established stem designs represent the same magnitude as the locally improved deviation to intact for SP-CL (Fig. 5). As those differences between CORAIL and CLS Spotorno may constitute measurable differences in remodeling and clinical outcome, the reduction of detrimental bone remodeling for anatomically shaped implants with grooves (sufficient surface area) and anatomic curvature such as the SP-CL should be expected in future studies (considering remodeling outcome) when compared to already successful implant stems. We are currently collecting evidence that the magnitude of those differences in strain-shielding that were calculated in this study lead to differences in clinical outcome. In the future, we plan to evaluate the longitudinal remodeling result in form of density changes as a consequence of the different local strain, validated with real patient follow-ups.

Using a shape optimization scheme based on a straight stem and varying the cross-sections, the proximally resorbed volume could not be reduced further than −23%^38^ or −21%^39^. Long-term proximal BMD-changes with this magnitude indicate that the limit of optimization for straight stem designs have been reached with the current stems. However, the mentioned approaches did not consider the combination of flexible stem, anatomic curvature and increased contact area (fit and fill). The conformity of the bone at the implant-bone interface and an extensive contact area have been neglected so far. However, those aspects of fit and fill are gaining more attention^40,41^. Inaba, *et al*.^42^ report lower BMD one year post-THA in Gruen zones 6 and 7 for a Zweymüller-type stem compared to a fit-and-fill-type stem. In Gruen zone 1, the fit-and-fill-type stem group showed a continuous decrease in BMD and the Zweymüller-type stem group showed a decrease in BMD up to 6 months after surgery and then showed an increase 12 months after surgery, which highlights the influence of later remodeling. Especially curved stems show improved fill which has to be considered alongside the well-regarded implant stiffness to achieve a more mechano-biologically adapted load transfer. The geometrical mismatch between the femoral canal and cementless implants should be met using more physiological stem designs that recreate the internal femoral shape, which has been shown to be more important than minimal cross-sectional area (or generally low implant stiffness) for physiological strain pattern and reduced stress shielding^37^. Especially the adaptation of stem choice to bone canal size and shape is important as a large tapered wedge-type stem and stovepipe femur may be associated with significant proximal BMD loss^42,43^. Undersized stems (canal fill index ≤80%) and stems in hips with cup revision were at higher risk for aseptic loosening with a hazard ratio of 4.2 and 4.3 respectively^2^. Inappropriate load transfer from implant to bone or inadequate internal load caused by excessive mal-positioning or inapt implant design (relative to canal size and shape) may cause this. The surgical access and according iatrogenic muscle trauma seem to play a lower-ranking role^3^.

Small changes in stem placement would likely have little influence on the internal loading of the femur after bony on-growth has been achieved and thus small positioning errors result in generally small strain differences when compared to the overall change from the intact femur strain^44^. This is confirmed by computational results suggesting that the strain distribution in the femur may be similar at different stages of healing after THA, regardless of small alterations in implant positioning. However, the healing immediately after surgery will be affected differently because the sensitivity of micro-motion is characteristic of the implant geometry^45^ and implant placement^46^. The sensitivity of stem design especially to initial micro-motion (vulnerability of on-growth) will have to be considered in future studies. In this study, we evaluated only implant geometry, however femur variability, especially Dorr femoral bone classification^47,48^, i.e. canal stovepipe shape versus champagne flute shape, should be included in future models of THA. Considering the range of anatomical parameters makes it possible to generalize or stratify the results to the entire population^42,43,45^. However, rather (intramedullary) femur shape than pure size seems to play the dominant role for strain-shielding^49^. The scope of this study was to test the influence of stem design features on bone strain. For consistency and control, we considered only one individual patient-specific geometry and its associated material distribution. However, in future studies markedly different geometries/properties are needed to see if these results about implant performance hold true across varying patient types. In our modeling approach, we did not realize a compaction which may locally condense bone^36^ and thus lead to a slightly different initial local strain, which might be especially relevant for the CORAIL with its special stepped surface and compaction broaching approach. We did not specifically validate the implant-bone interface behavior, quantify possible wear or consider particle-induced inflammation here as we assumed uneventful bony on-growth. We did not model any initial pressure between bone and implant or viscoelastic behavior. Further experimental measurements are required to validate the results of the FE model.

## Conclusions

This study indicates that small changes in geometry of uncemented stems can change strain-shielding considerably up to 20% locally. Combinations of moderately low stem stiffness (slender titanium stem with deep grooves) and a large proximal contact area (anatomically curved stem in combination with large surfaces through ribs or grooves) lead to reduced strain-shielding, estimated through finite element analyses.

## Outlook

Insights to long-term effects of the improved strain-shielding on bone mass can be gained by clinical studies and may eventually be explained by mathematical remodeling analyses. Both are parts of our ongoing research activities.

## Data Availability

The datasets generated during and/or analysed during the current study excluding protected intellectual and commercial property are available from the corresponding author on reasonable request.
